# Hydrogen sulfide as a therapeutic agent for diabetic wounds: effects on inflammation and fibroblast pyroptosis

**DOI:** 10.3389/fimmu.2025.1558443

**Published:** 2025-08-27

**Authors:** Fusheng Zhao, Yuanyuan Li, Qunying Hu, Jiali Xu, Na Zhang, Yonglan Chen, Xinyue Jiang, Chunfu Gu, Kexin Zhang, Geng Wu

**Affiliations:** ^1^ Department of Histology and Embryology, Mudanjiang Medical University, Mudanjiang, China; ^2^ Department of Biochemistry and Molecular Biology, Mudanjiang Medical University, Mudanjiang, China

**Keywords:** hydrogen sulfide, diabetic skin wound healing, skin fibroblast, NLRP3 inflammasome, pyroptosis, NF-κB pathway

## Abstract

**Introduction:**

Chronic nonhealing wounds are one of the most serious complications of diabetes mellitus (DM), with limited treatment options. Hydrogen sulfide (H_2_S) plays a protective role against multiple inflammatory diseases. This study aimed to explore the effects of H_2_S on diabetic skin wound healing and its underlying mechanisms.

**Methods:**

A streptozotocin-induced diabetic rat model was established, and the rats were randomly divided into control, DM, and DM + NaHS (a donor of H_2_S) groups. Full-thickness wounds were made on the dorsal skin of the rats. H_2_S levels and H_2_S-synthesizing enzyme expression were evaluated in the wound tissue. Wound healing, histological changes, inflammasome activation, fibroblast pyroptosis, and phosphorylation of signaling components of nuclear factor kappa B (NF-κB) pathway were assessed.

**Results:**

The results showed that NaHS administration effectively restored H_2_S levels and promoted skin wound healing, as evidenced by the amelioration of histological changes and increased collagen deposition in diabetic rats. Meanwhile, NaHS treatment inhibited macrophage M1 polarization and decreased the levels of pro-inflammatory cytokines, such as tumor necrosis factor-α (TNF-α), interleukin-1β (IL-1β), and interleukin-6 (IL-6) in diabetic wound tissues, notably, suppressing NOD-like receptor family pyrin domain-containing 3 (NLRP3) inflammasome activation and fibroblast pyroptosis. In addition, NaHS treatment was able to inhibit the activation of NF-κB pathway in the wound tissues.

**Conclusion:**

Taken together, these results show that H_2_S promotes skin wound healing in diabetic rats and may be involved in the restoration of H_2_S levels, inhibition of NLRP3 inflammasome activation, and fibroblast pyroptosis, suggesting that it may be a promising therapeutic agent for treating diabetic skin wounds.

## 1 Introduction

According to recent data from the International Diabetes Federation, as of 2021, it is estimated that over 537 million people worldwide have been diagnosed with diabetes mellitus (DM); this number is expected to reach 643 million by 2030, and 783 million by 2045 (1). Chronic nonhealing skin wounds are one of the most serious complications of DM, adversely affecting the quality of life of patients and even leading to disability and amputation. Unfortunately, no effective treatments are currently available for this devastating disease. Therefore, there is an urgent need to identify novel candidate agents or therapies that promote wound healing in the skin of diabetic patients.

Wound healing is a dynamic and interactive process that involves an orchestrated cascade of events including hemostasis, inflammation, cell proliferation, and remodeling (2). Upon injury, monocytes infiltrate the wound site and differentiate into pro-inflammatory macrophages (M1-like phenotype) and anti-inflammatory macrophages (M2-like phenotype) in response to wound microenvironmental cues. The M1-like macrophages produce various pro-inflammatory cytokines, including the tumor necrosis factor-α (TNF-α), interleukin-1β (IL-1β), and interleukin-6 (IL-6) (3). Conversely, M2-like macrophages secrete anti-inflammatory cytokines and growth factors that promote angiogenesis and wound healing. Therefore, M1 macrophage polarization and inflammatory response suppression is essential for wound healing. Multifunctional hydrogels reportedly alleviate inflammation and accelerate chronic diabetic skin wound healing significantly with the ability to control therapeutic substance release according to changes in the wound microenvironment (4, 5). Accumulating evidence suggests that poor healing of diabetic wound may be attributed to aberrant activation of nuclear factor kappa B (NF-κB) signaling pathway and NOD-like receptor family pyrin domain-containing 3 (NLRP3) inflammasome (6). The NF-κB signaling pathway, a pro-inflammatory pathway, regulates the transcription of NLRP3 genes, and triggers activation of caspase-1 that cleaves pro-inflammatory factors pro-IL-1β and pro-interleukin-18 (IL-18) to mature (IL-1β and IL-18) forms, which mediate inflammatory responses and lead to cell pyroptosis (7). The NLRP3 inflammasome is an essential sensor in the innate immune system and consists of the sensor protein NLRP3, an adapter protein known as apoptosis-associated speck-like protein containing CARD (ASC), and procaspase-1 (8). The aberrant activation of NF-κB pathway along with NLRP3 inflammasome was found to be related to a wide variety of inflammatory diseases (6). A previous study demonstrated that neutrophil extracellular traps induce NLRP3 inflammasome activation and inflammation in diabetic wounds (9). A recent study demonstrated that reactive oxygen species (ROS)-activated NLRP3 inflammasomes hamper diabetic wound healing (10). Several studies have indicated that activation of the NLRP3 inflammasome can drive pyroptosis in Schwann cells (11), hepatocytes (12), and podocytes (13). However, whether NLRP3 inflammasome is involved in diabetes-induced fibroblast pyroptosis in diabetic wound tissues remains unclear.

Hydrogen sulfide (H_2_S), a novel gasotransmitter, is synthesized from L-cysteine and/or L-homocysteine by the cystathionine-γ-lyase (CSE), cystathionine-β-synthase (CBS), and 3-mercaptopyruvate sulfurtransferase (3-MST) in mammals. H_2_S plays crucial roles in the regulation of various physiological functions and exhibits anti-inflammatory activity by affecting NLRP3 inflammasome activation in many diseases such as high choline-induced cardiac dysfunction (14), ischemia–reperfusion-induced kidney injury (15), and renal ischemia–reperfusion-induced lung injury (16). It has been reported that H_2_S attenuated high glucose-induced cardiotoxicity by suppressing the activation of the NLRP3 inflammasome mediated by the toll-like receptor 4/NF-κB pathway (17). Additionally, H_2_S was able to alleviate inflammation in dextran sulfate sodium (DSS)-induced colitis via anti-inflammatory mechanisms, including inhibition of NF-κB signaling (18). These observations led us to hypothesize that H_2_S would regulate NLRP3 inflammasome activation and fibroblast pyroptosis in diabetic wound tissues. Thus, the aim of the present study was to investigate whether H_2_S could inhibit NLRP3 inflammasome activation, attenuate the inflammatory response in wound tissues, and ultimately promote diabetic skin wound healing.

## 2 Materials and methods

### 2.1 Animals and experimental groups

Forty-eight male Sprague-Dawley rats (8-weeks old; weighing 250 ± 10 g) were purchased from Liaoning Chang-Sheng Biotech Co., Ltd. (Benxi, China). Rats were housed in cages under pathogen-free conditions and a 12 h light/dark cycle, at 22°C and 30%–40% humidity. They had free access to food and water. Following acclimation for 1 week, the rats were randomly allocated into control, DM, and DM + NaHS groups using computer-generated randomization sequences. All animal experiments complied with the Guidelines for the Care and Use of Laboratory Animals published by the US National Institutes of Health (1996) and were approved by the Animal Protection and Use Committee of Mudanjiang Medical University.

### 2.2 Establishment of a full-thickness skin excisional wound

Diabetes was induced by injecting streptozotocin (STZ) as described previously (19). Rats in the DM group were deprived of food for 18 h before intraperitoneal injection of 1% STZ (50 mg/kg; Sigma-Aldrich, St. Louis, MO, USA). Rats with blood glucose levels > 16.67 mmol/L after three days were considered diabetic. Three weeks after STZ induction, diabetic rats were anesthetized by intraperitoneal injection of pentobarbital (35 mg/kg), and a circular full-thickness skin excisional wound (2 cm in diameter) was generated on the shaved dorsum of the rats as previously described (20). In addition to the surgery described above, rats in the DM + NaHS group were administered freshly prepared NaHS (a donor of H_2_S) solution (56 mmol/kg) by intraperitoneal injection once a day for 21 consecutive days, based on a previous study (21). The rats in the control and DM groups received equivalent volumes of normal saline daily. To validate the anti-inflammatory effect of NaHS, we used the NLRP3 inflammasome inhibitor MCC950 as a comparative agent. We randomly assigned a second cohort of rats into five groups (n = 9 per group) as follows: control, DM, DM + NaHS, DM + MCC950, and DM + MCC950 + NaHS. We dissolved MCC950 (Sigma-Aldrich, St. Louis, MO, USA) in normal saline and administered it daily via intraperitoneal injection at a dose of 10 mg/kg body weight, based on previously established protocols (22). Body weight and blood glucose levels of the rats were monitored throughout the experimental period.

### 2.3 H_2_S level measurement

The levels of H_2_S in injured tissues composed of the wound bed and surrounding skin were detected on day 21 after surgery using a 7-azido-4-methylcoumarin (C-7Az; Sigma, USA) fluorescence probe. Briefly, frozen sections were incubated with 50 μM C-7Az solution at 37°C for 1 h in the dark, followed by washing three times with phosphate-buffered saline (PBS). Fluorescence images of C-7Az were acquired using a fluorescence microscope (Nikon, Japan), and the fluorescence intensity was analyzed using Image Pro Plus software (version 6.0; Media Cybernetics, Rockville, MD, USA). The level of H_2_S in skin samples was measured using an H_2_S testing kit (Nanjing Institute of Bioengineering, Nanjing, China) according to the manufacturer’s instructions. The level of H_2_S is expressed as nmol/mg protein. Protein concentrations were determined using a bicinchoninic acid (BCA) protein assay kit (Beyotime, Shanghai, China).

### 2.4 Wound healing assay

Wound healing was evaluated as previously described (23). Briefly, skin wounds were photographed by an observer blind to the grouping on days 0, 7, 14, and 21 postoperatively. The wound areas were measured using the ImageJ software (National Institutes of Health, Bethesda, MD, USA), and the wound closure rate was calculated independently by two investigators blind to the grouping. Wound closure rate (%) = (initial wound area on day 0 −wound area at different time points)/initial wound area on day 0 × 100.

### 2.5 Blood flow measurement

We assessed the blood flow in the wound area using a portable Esaote MylabX7 ultrasound scanner equipped with a 10 MHz linear probe (Esaote SpA, Genova, Italy) on post-surgery days 7, 14, and 21. Briefly, we anesthetized the rats with pentobarbital and fastened them to the operating table. We applied a layer of ultrasound coupling agent to the skin and positioned the probe perpendicular to the skin surface for imaging. The same sonographer performed all examinations.

### 2.6 Histological assessment

On day 21 after surgery, the rats were sacrificed and the skin tissues along the outer edge of the wound and the wound beds were isolated and fixed in 4% paraformaldehyde for 48 h. Subsequently, the wounded tissues were dehydrated in a graded alcohol series, cleaned in xylene, embedded in paraffin, and sectioned into 5-μm-thick sections. Following staining with hematoxylin-eosin (HE) and Masson’s trichrome stains, the sections were observed and photographed using a light microscope (Olympus, Japan). The length of the wound area was measured blindly by one person using ImageJ software, in accordance with a previous report (24). Masson’s trichrome staining was used to detect collagen deposition in the wound tissue, and the mean staining intensity was analyzed in three visual fields randomly selected per section using Image-Pro Plus 6 software. The analysis was performed by an investigator who was blinded to the experimental grouping.

### 2.7 Transmission electron microscopy examination

On day 21 after surgery, skin samples were collected, fixed in 2.5% glutaraldehyde, and sectioned using a diamond knife on copper grids. The grids were examined under an electron microscope (Hitachi, Tokyo, Japan).

### 2.8 Immunofluorescence analysis

On day 21 after surgery, skin samples were collected and snap-frozen in optimal cutting temperature compound to prepare frozen sections (10 μm). After fixing in 4% paraformaldehyde, the sections were blocked with 5% goat serum in PBS for 1 h, and then incubated overnight at 4°C using the following primary antibodies: collagen I (1:300), collagen II (1:100), collagen III (1:300), and collagen IV (1:100) obtained from Bioss, Beijing, China; nitric oxide synthase (iNOS; 1:100), CD206 (1:200), CD68 (1:200), CD80 (1:300), vimentin (1:500), ASC (1:200), NLRP3 (1:100), caspase-1 (1:100), gasdermin D (GSDMD; 1:100) and IL-18 (1:200), obtained from Proteintech, Wuhan, China. The sections were then incubated with second antibodies at 37°C for 2 h, followed by the 4′,6-diamidino-2-phenylindole (DAPI; 5μg/ml; Beyotime) nuclear staining. Images were acquired using a fluorescence microscope (Olympus).

### 2.9 Cytokine measurement

Skin wound tissues of each group were homogenized in cold PBS containing protease inhibitor cocktail, and centrifuged at 10,000 rpm for 10 min at 4°C. Then, the supernatants were collected to measure the levels of TNF-α, IL-1β, and IL-6 using enzyme-linked immunosorbent assay kits according to the manufacturer’s instructions. The protein concentrations of all the samples were determined using a BCA protein assay kit (Beyotime).

### 2.10 Western blotting analysis

Skin samples were lysed in radioimmunoprecipitation assay lysis buffer, and protein concentration was determined using a BCA protein assay kit. Equal amounts of protein (30 μg) were separated by 10% sodium dodecyl sulphate-polyacrylamide gel electrophoresis, and transferred to a polyvinylidene difluoride membrane (Millipore, MA, USA), and the blots were blocked with 5% skimmed milk in Tris-buffered saline (10 mM Tris-HCl pH 7.5, 150 mM NaCl) containing 0.1% Tween-20 (TBST) for 1 h. Then, the membranes were incubated with primary antibodies at 4°C overnight, followed by incubation with secondary antibodies at 37°C for 2 h. The following antibodies were used: collagen I (1:1000), collagen II (1:1000), collagen III (1:2000), collagen IV (1:1000), CSE (1:1000), CBS (1:1500), 3-MST (1:1000), iNOS (1:1000), CD206 (1:2000), CD80 (1:1500), NLRP3 (1:1000), ASC (1:1000), cleaved caspase-1 (1:1000), IL-18 (1:1200), and GSDMD-N (1:100; Bioss). After washing three times with TBST, the membranes were incubated with a secondary antibody (1:5000; Proteintech). Immunoreactive bands were identified using enhanced chemiluminescence (Thermo Fisher Scientific, Waltham, USA), and imaged using a ChemiDoc XRS Plus luminescent imaging system (Bio-Rad, Hercules, CA, USA). Densitometric quantification of relative band intensities was performed using Image-Pro Plus 6.0, and the levels of target proteins were normalized to the band of β-actin.

### 2.11 Statistical analysis

All data are presented as mean ± standard deviation (SD). Statistical analyses were performed using the SPSS software (version 18.0; SPSS Inc., Chicago, IL, USA). Statistical comparisons among multiple groups were performed using one-way analysis of variance followed by Tukey’s multiple comparison test. Statistical significance was set at *P* < 0.05.

## 3 Results

### 3.1 Blood glucose levels and body mass

To exclude a reduction in blood glucose as a potential mechanism accelerating skin wound healing, glucose levels were monitored at 0, 3, 7, 14, and 21 days after STZ injection. The blood glucose levels of rats in the DM and DM + NaHS groups markedly increased three days after STZ injection, and there was no statistical difference between the DM and DM + NaHS groups (**Table 1**). The body weight of the rats in the DM group was significantly lower than that of the rats in the control group. In addition, differences in mean body weight between the DM and DM + NaHS groups were not statistically significant (**Table 2**). These results indicated that the DM model of STZ injection was well established in this study.

**Table 1:** Blood glucose levels of rats in each group (mmol/L).

Group	Before STZ injection	Day 3 post-STZ injection	Day 7 post-STZ injection	Day 14 post-STZ injection	Day 21 post-STZ injection
control	4.72 ± 0.13	4.73 ± 0.16	4.80 ± 0.11	4.85 ± 0.17	4.78 ± 0.10
DM	4.77 ± 0.12	28.22 ± 0.24*	28.26 ± 0.4*	28.06 ± 0.14*	27.98 ± 0.32*
DM + NaHS	4.72 ± 0.11	28.13 ± 0.26*	28.15 ± 0.28*	27.87 ± 0.46*	28.08 ± 0.41*

*P < 0.05 vs. control group.

**Table 2:** Body weights of rats in each group (g).

Group	Before STZ injection	Day 3 post-STZ injection	Day 7 post-STZ injection	Day 14 post-STZ injection	Day 21 post-STZ injection
control	258.52 ± 1.67	268.67 ± 4.41	294.75 ± 5.08	324.41 ± 3.30	348.68 ± 7.22
DM	257.02 ± 2.93	261.27 ± 3.69*	278.81 ± 4.32*	304.11 ± 7.84*	288.18 ± 5.19*
DM + NaHS	257.28 ± 1.70	265.33 ± 3.88*	278.40 ± 1.80*	303.08 ± 8.16*	287.13 ± 8.93*

*P < 0.05 vs. control group.

### 3.2 Effects of NaHS on H_2_S levels and H_2_S-synthesizing enzyme expression in skin wound tissues

**Figure 1:**
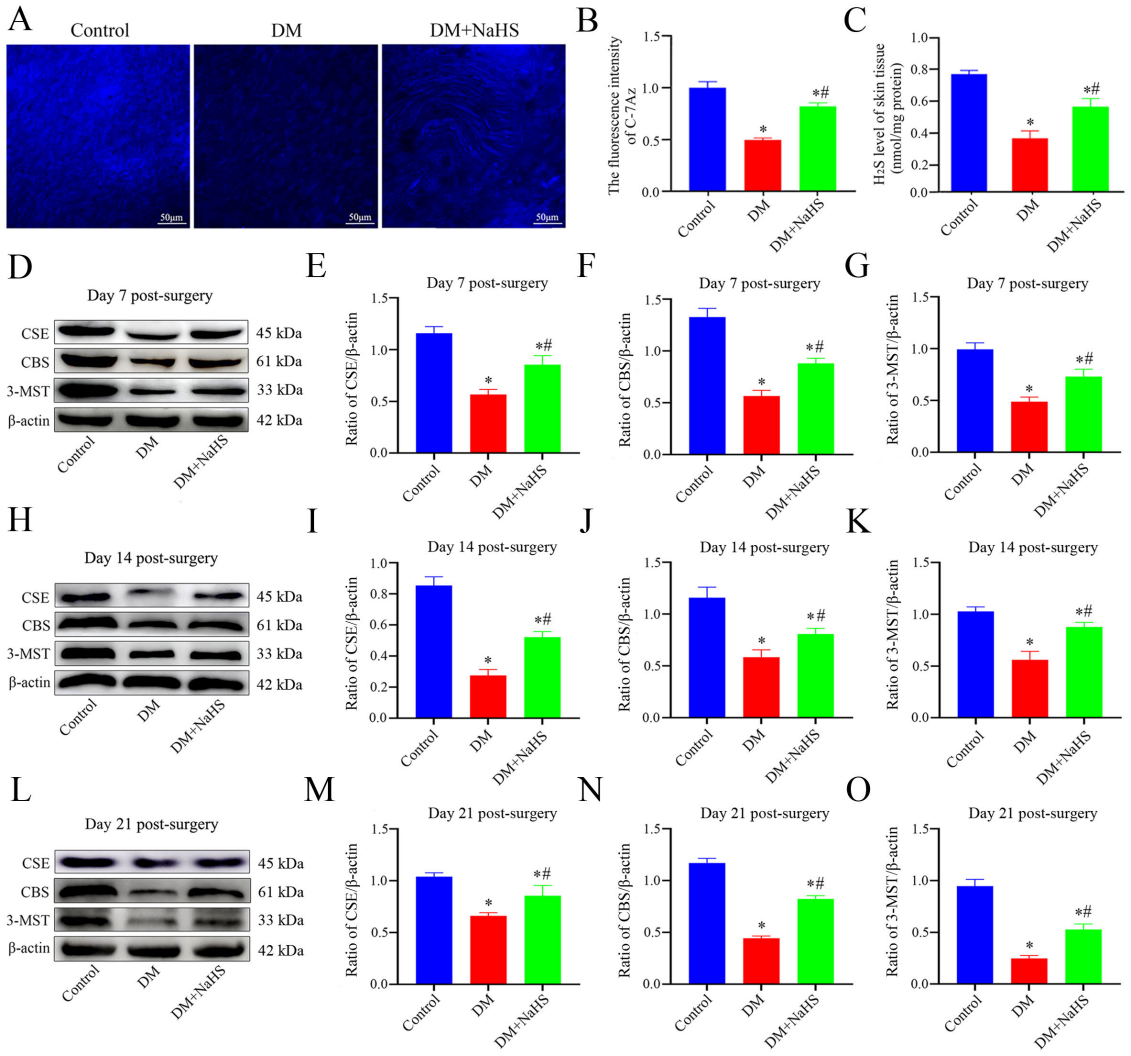
Evaluation of the levels of H_2_S and H_2_S-synthesizing enzymes expression in rat skin wound tissue. **(A)** Representative images of the skin wound tissues stained with a C-7Az fluorescence probe on day 21 postoperatively. **(B)** Quantification of C-7Az fluorescence intensities in the control, DM, and DM + NaHS groups. **(C)** The levels of H_2_S in skin wound tissues of the control, DM, and DM + NaHS groups on day 21 postoperatively. **(D–O)** Representative western blots and quantitative analysis of protein levels of CSE, CBS, and 3-MST in skin wound tissues of the control, DM and DM + NaHS groups on days 7, 14, and 21 postoperatively. Scale bar: 50 µm. *P < 0.05 vs. control group; ^#^ *P* < 0.05 vs. DM group; n = 6.

To evaluate the effects of STZ and NaHS on H_2_S levels in the wound tissues, C-7Az, a specific H_2_S fluorescent probe, was used, and the results are shown in **Figure 1A**. Skin samples from the DM group produce lower levels of H_2_S, and a weak fluorescence signal. H_2_S levels were elevated by NaHS treatment, which led to a more intense fluorescence signal than that in the DM group. H_2_S levels were quantified based on the fluorescence intensity of C-7Az. As shown in **Figure 1B**, the fluorescence intensity of C-7Az in the DM group was significantly lower than that in the control group (*P* < 0.05). The level of H_2_S in the DM + NaHS group was enhanced compared to that in the DM group (*P* < 0.05) but did not recover to the level of the control group (*P* < 0.05). Similarly, the results showed that H_2_S levels distinctly decreased in the DM group. However, the decrease in H_2_S levels was elevated following NaHS administration, which was in agreement with the results of C-7Az detection (**Figure 1C**). In addition, H_2_S-synthesizing enzyme expression was significantly reduced in the DM group compared to the control group on post-surgery days 7, 14, and 21 (*P* < 0.05). Administration of NaHS, however, increased the levels of H_2_S-synthesizing enzymes in the DM + NaHS group, which were higher than those in the DM group (*P* < 0.05) (**Figures 1D–O**). These results suggest that NaHS can restore H_2_S levels in skin wound tissues of diabetic rats.

### 3.3 Effect of NaHS on skin wound healing in diabetic rats

**Figure 2:**
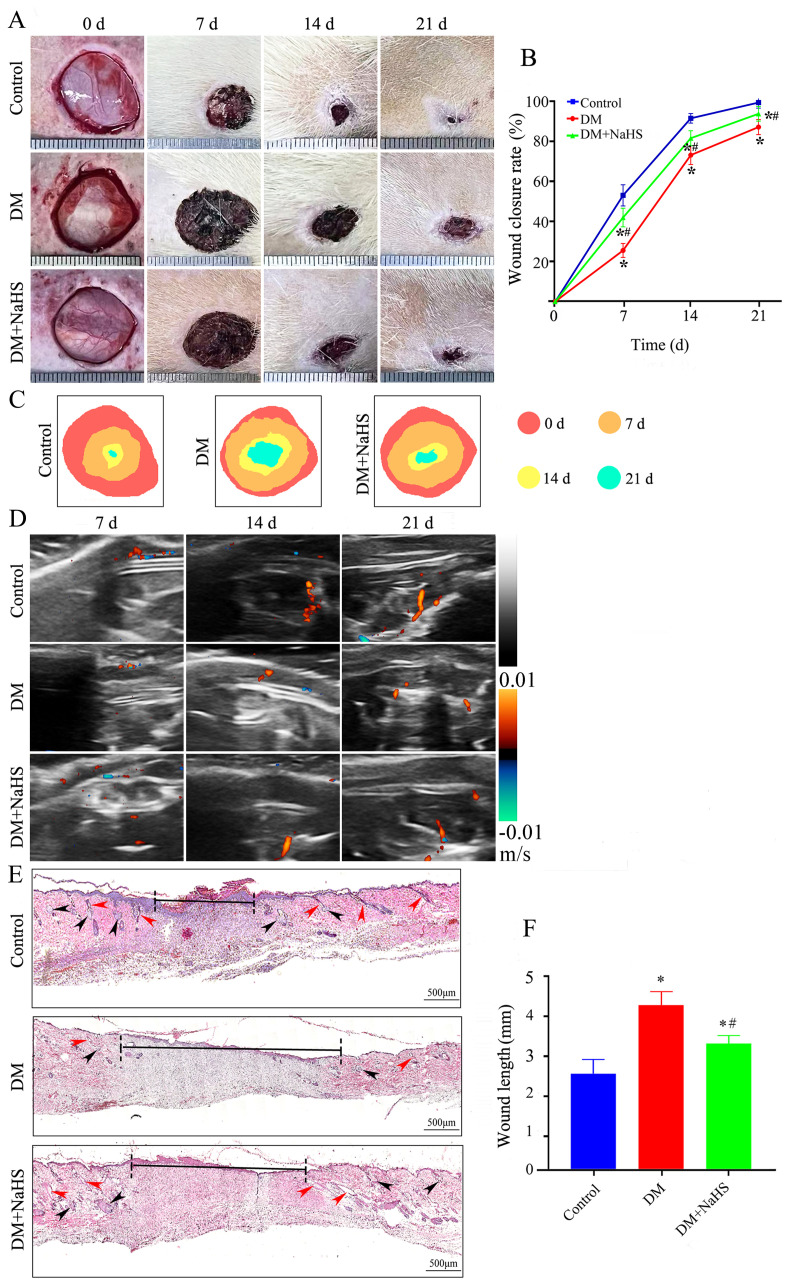
Administration of NaHS promotes skin wound healing in diabetic rats. **(A)** Gross view of skin wounds in the control, DM, and DM + NaHS groups on days 0, 7, 14, and 21 postoperatively. **(B)** Wound closure rates at different time points in the control, DM, and DM + NaHS groups. **(C)** Wound healing trajectories on postoperative days 0, 7, 14, and 21 in the control, DM, and DM + NaHS groups. **(D)** Representative Doppler images of blood perfusion 7, 14, and 21 days after surgery. Blue and red colors represent low and high blood flow, respectively. **(E)** Representative images of HE-stained sections of skin wound tissues showing typical histological changes 21 days postoperatively in the control, DM, and DM + NaHS groups. **(F)** Quantification of the wound width 21 days postoperatively in the control, DM, and DM + NaHS groups. The red and black arrows indicate the hair follicles and sebaceous glands, respectively. *P < 0.05 vs. control group; ^#^ *P* < 0.05 vs. DM group; n= 6.

To investigate the effect of NaHS on wound healing, diabetic rats were treated with NaHS for 21 days after full-thickness wounds were created in their dorsal skin. As shown in **Figures 2A–C**, the wound closure rate of the NaHS-treated rats was accelerated, as illustrated by the smaller wound areas at 7, 14, and 21 days post-surgery compared to the DM rats (*P* < 0.05). To further evaluate the effect of NaHS on skin wound healing, we assessed blood perfusion in the wound area. Doppler blood flow analysis indicated a marked increase in the blood flow around the wound in the DM + NaHS group, on post-surgery days 7, 14, and 21, compared to the DM group (**Figure 2D**). Histological changes in wound tissues were examined on post-surgery day 21. As shown in **Figure 2E**, along with enhanced re-epithelialization, NaHS treatment accelerated the regeneration of hair follicles, sebaceous glands, and connective tissues in the wound sites of diabetic rats. Quantitative analysis showed that the length of the wound area in the DM group was significantly longer than that in the control group (*P* < 0.05); however, the length of the wound area was significantly reduced in the DM + NaHS group compared to that in the DM group on day 21 post-surgery (*P* < 0.05) (**Figure 2F**). These results indicate that the administration of NaHS increased wound blood supply and promoted skin wound healing in diabetic rats.

### 3.4 Effect of NaHS on collagen deposition in the skin wound tissues of diabetic rats

**Figure 3:**
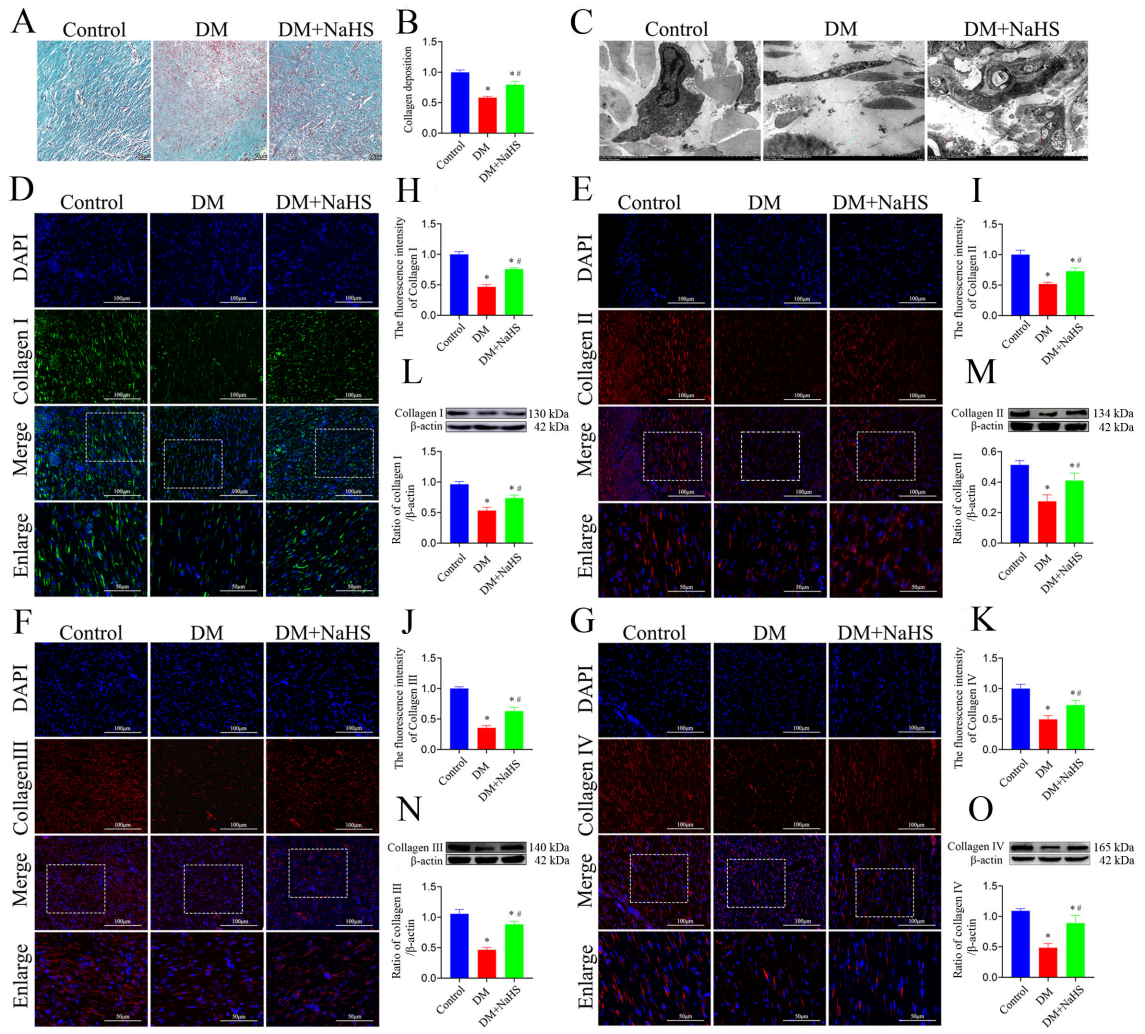
NaHS promotes collagen deposition in skin wound tissues of diabetic rats. **(A)** Representative images of Masson’s trichrome-stained sections 21 days postoperatively in the control, DM, and DM + NaHS groups. **(B)** Quantification of blue granulation intensity within wound sites. **(C)** Representative TEM images of skin wound tissues from the control, DM, and DM + NaHS groups 21 days postoperatively. **(D–K)** Representative immunofluorescence images of collagen I, II, III, and IV and fluorescence intensity analysis in the control, DM, and DM + NaHS groups 21 days post-surgery. **(L-O)** Representative western blots and quantitative analysis of protein levels of collagen I, II, III, and IV in skin wound tissues of the control, DM, and DM + NaHS groups 21 days postoperatively. Scale bar (Masson’s staining images): 50 µm; (immunofluorescence staining images): 100 µm; (TEM images): 0.5 μm. *P < 0.05 vs. control group; ^#^ *P* < 0.05 vs. DM group; n = 6.

To confirm whether NaHS promotes collagen deposition at wound sites, Masson’s trichrome staining was performed. As shown in **Figure 3A**, large numbers of neatly arranged collagen fibers were observed in the wound tissues of the DM + NaHS group, whereas collagen fibers were rarely found in the DM group. Quantification of the intensities of the stained areas revealed that collagen deposition in the wounds of the DM + NaHS group was markedly higher than that in the DM group (**Figure 3B**). To further test the effect of NaHS on ultrastructural alterations in skin wound sites, TEM was performed. As shown in **Figure 3C**, collagen fibers were sparse and irregularly arranged in the wound sites of diabetic rats, whereas wavy collagen fibers were abundant in the wound tissues of diabetic rats treated with NaHS. The morphology of the collagen fibers and fibroblast structures was normal in the control group. Collagen deposition at the wound sites was detected by immunofluorescence staining and western blotting. The results showed that the administration of NaHS distinctly increased the amount of collagen in the wound tissue, and much more collagen deposition was observed at the wound sites in the DM + NaHS group than in the DM group (**Figures 3D–K**). Quantification of protein expression revealed that the levels of collagen I, II, III, and IV in the wound tissues of the DM + NaHS group were significantly higher than those in the DM group (*P* < 0.05), which did not reach the levels found in the control group (*P* < 0.05; **Figures 3L–O**). These results suggest that NaHS promotes collagen deposition in the skin wound tissues of diabetic rats.

### 3.5 Effect of NaHS on the inflammatory response in skin wound tissues of diabetic rats

**Figure 4:**
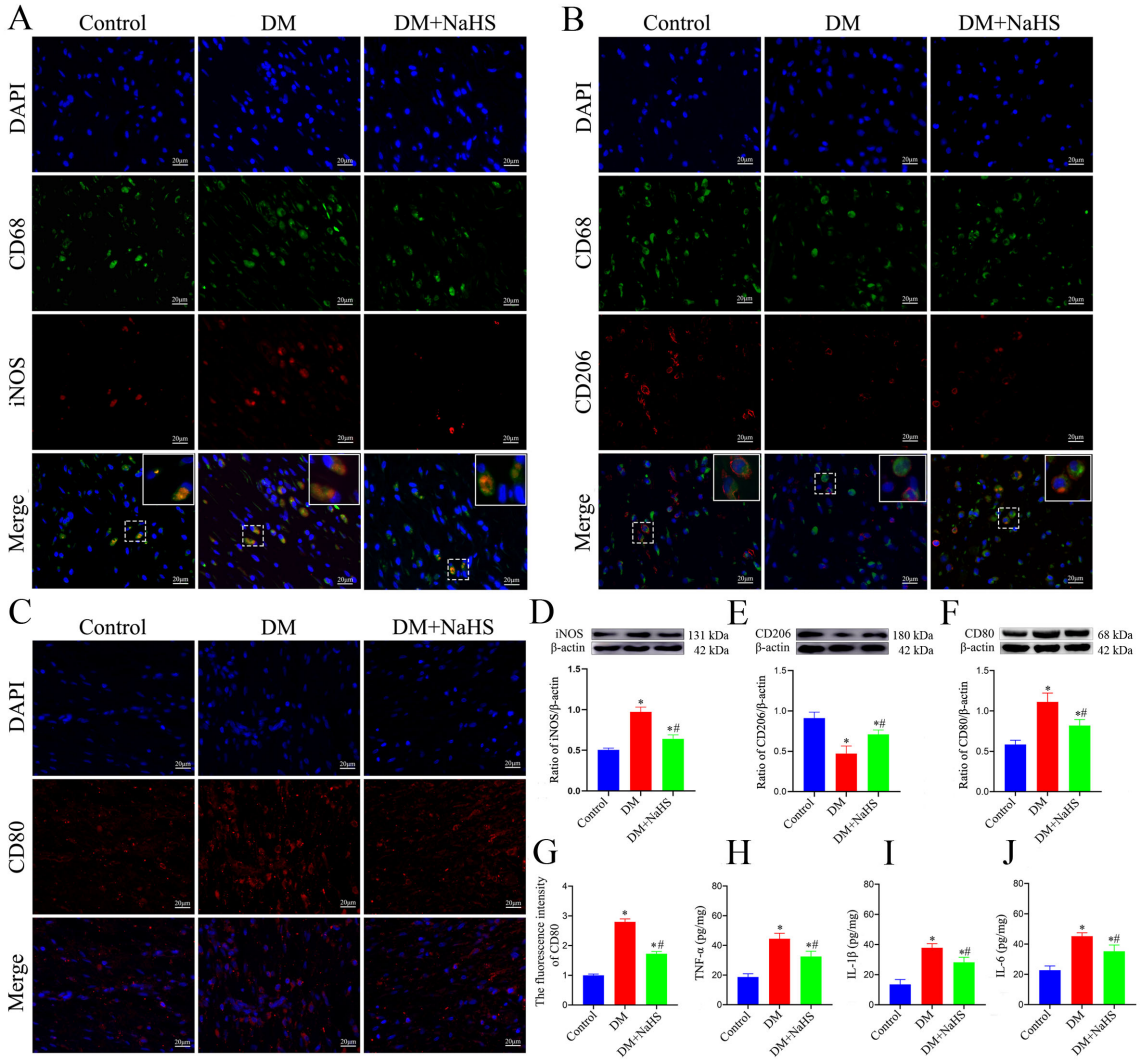
NaHS alleviated inflammation in skin wound tissues of diabetic rats. **(A, B)**Representative immunofluorescent images of M1-and M2-like macrophages stained with CD68/iNOS and CD68/CD206, respectively, in skin wound tissues of the control, DM, and DM + NaHS groups 21 days postoperatively. Scale bar: 20 mm. **(C, G)** Representative immunofluorescent images of M1-like macrophages stained with CD80 and fluorescence intensity analysis in the control, DM, and DM + NaHS groups 21 days postoperatively. Scale bar: 20 μm. **(D−F)** Representative western blots and quantitative analysis of iNOS, CD206, and CD80 protein levels in skin wound tissues of the control, DM, and DM + NaHS groups 21 days postoperatively. **(H−J)** Quantitative analysis of TNF-α, IL-1β, and IL-6 levels in skin wound tissues of the control, DM, and DM + NaHS groups 21 days postoperatively. *P < 0.05 vs. control group; ^#^ *P* < 0.05 vs. DM group; n = 6.

To evaluate the effects of NaHS on inflammation in skin wound tissues, M1- and M2-like macrophages were detected by double-immunofluorescence staining. The M1 phenotype was assessed using specific markers (CD68/iNOS), and the results indicated that the number of CD68^+^iNOS^+^ double-positive macrophages was markedly increased at the wound sites in diabetic rats. Similarly, iNOS protein expression was higher in the DM group than in the DM + NaHS group (**Figures 4A, D**). In parallel, the M2 phenotype was evaluated using CD68/CD206 staining. It was found that the M2-like macrophages substantially decreased in wound sites of the diabetic rats. However, the administration of NaHS significantly increased the number of M2-like macrophages in skin wound tissues (**Figures 4B, E**). Moreover, we applied the M1-like macrophages marker (CD80) to corroborate the effect of NaHS on macrophages polarization. Our result showed that NaHS treatment effectively reduced CD80 protein expression in diabetic wound tissues, consistent with the results of the immunofluorescence staining (**Figures 4C, G, F**). Accordingly, the inflammatory cytokines TNF-α, IL-1β and IL-6 were also measured in skin wound tissues, which showed that their levels increased in the DM group, but decreased with NaHS treatment (**Figures 4H–J**). Taken together, these results suggest that NaHS can reduce the population of M1-like macrophages, increase the population of M2-like macrophages, and suppress the release of proinflammatory cytokines in the skin wound tissues of diabetic rats.

### 3.6 Effect of NaHS on inflammasome activation in the skin fibroblasts of diabetic rats

**Figure 5:**
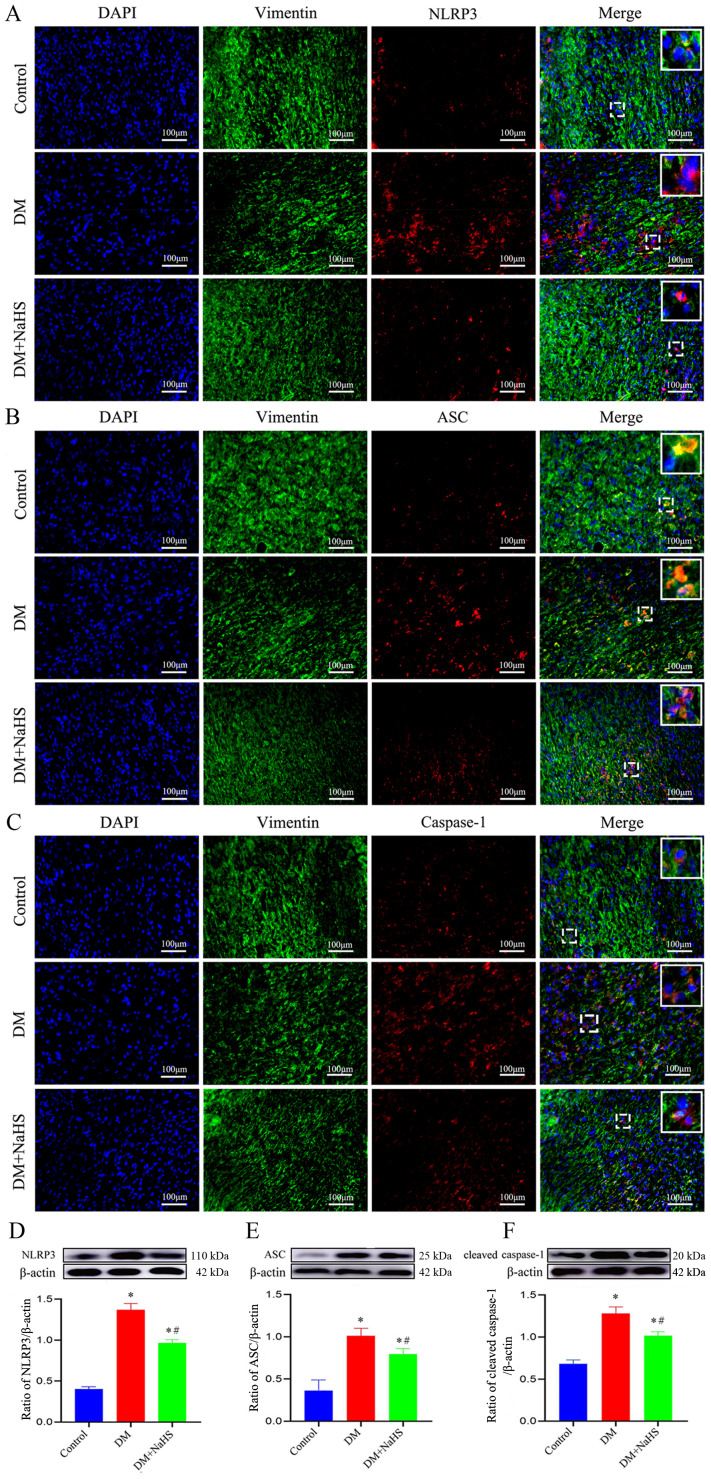
NaHS suppresses inflammasome activation in skin fibroblasts of diabetic rats. **(A-C)** Representative immunofluorescent images of the colocalization of vimentin (a fibroblast marker) with NLRP3, ASC, and caspase-1 in skin fibroblasts 21 days postoperatively. Scale bar: 100 µm. **(D-F)** Representative western blots and quantitative analysis of the protein levels of NLRP3, ASC, and cleaved caspase-1 in the skin wound tissues of the control, DM, and DM + NaHS groups 21 days postoperatively. *P < 0.05 vs. control group; ^#^ *P* < 0.05 vs. DM group; n = 6.

**Figure 6:**
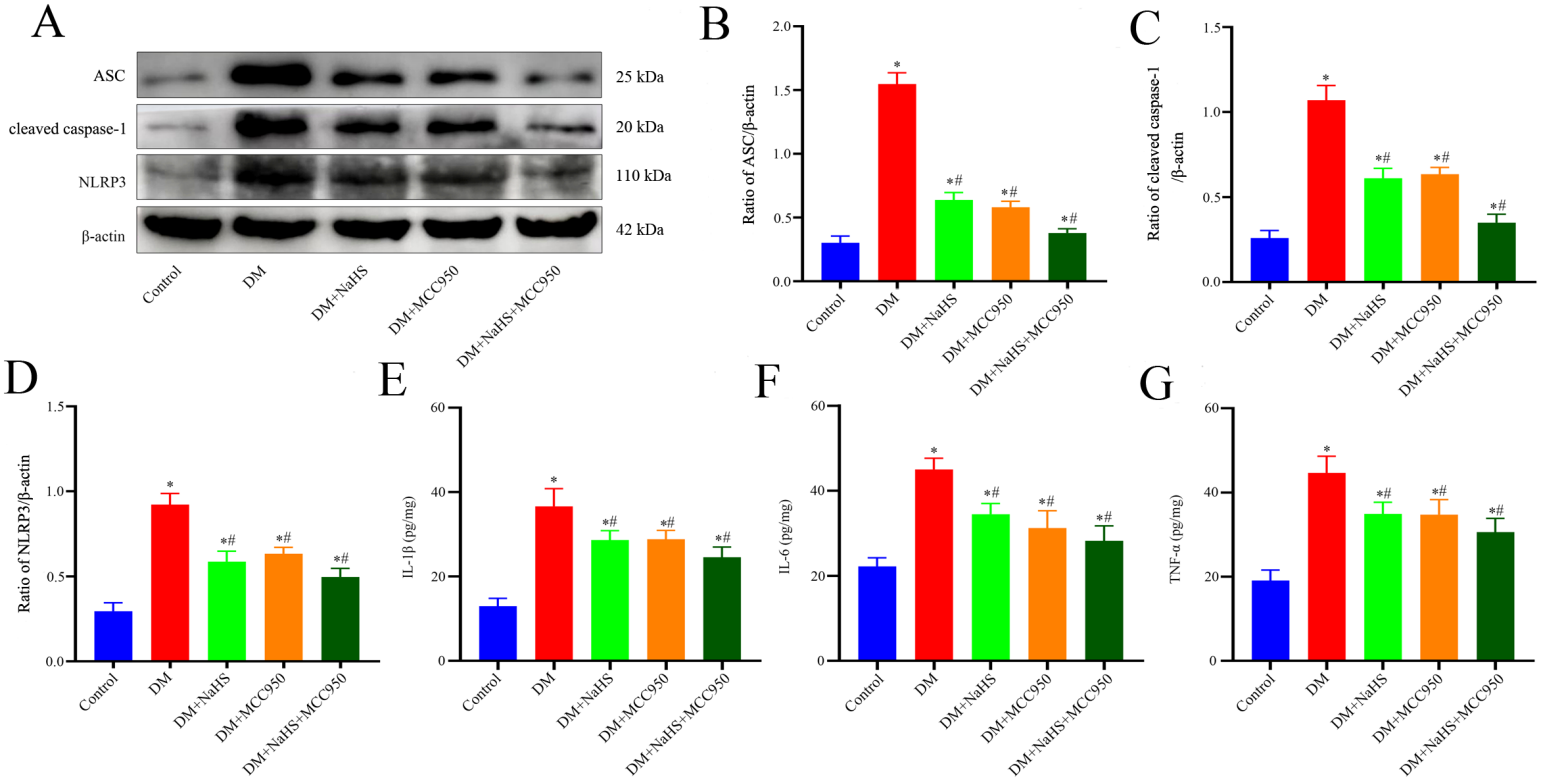
The MCC950 and NaHS treatments reduced NLRP3-associated protein and inflammatory factor expression levels in diabetic wound tissues. **(A-D)** Representative western blots and quantitative analysis of ASC, cleaved caspase-1, and NLRP3 protein expressions in skin wound tissues 21 days postoperatively. **(E-G)** Quantitative analysis of IL-1β, IL-6, and TNF-α levels in skin wound tissues of the control, DM, DM + NaHS, DM + MCC950, and DM + MCC950 + NaHS groups 21 days postoperatively. *P < 0.05 vs. control group; ^#^ *P* < 0.05 vs. DM group; n = 6.

To determine whether H_2_S is involved in the regulation of inflammasome activation in fibroblasts, double immunofluorescence staining was performed to detect the protein expression of NLRP3, ASC, and caspase-1 in skin fibroblasts at days 21 post-injury. As shown in **Figures 5A–C**, the protein expression of NLRP3, ASC, and caspase-1 in fibroblasts within the skin wound sites of diabetic rats was significantly increased compared to levels in the fibroblasts of the control group (*P* < 0.05). However, these increases were abrogated by NaHS treatment. Similarly, western blotting showed that the protein expression levels of NLRP3, ASC, and cleaved caspase-1 were substantially increased in the wound tissues of the DM group compared with those in the control group (*P* < 0.05), whereas the expression levels of all these proteins were distinctly reduced in the DM + NaHS group (**Figures 5D–F**). To further corroborate how NaHS affects NLRP3 inflammasome activation, we used MCC950, a specific NLRP3 inhibitor, as a comparative agent. Our results revealed that the levels of NLRP3 inflammasome components, NLRP3, ASC, and cleaved caspase-1, significantly decreased in the MCC950-treated group relative to the DM group (*P* < 0.05). Similarly, the expression of these proteins was reduced in the NaHS-treated group. In addition, TNF−α, IL−1β, and IL−6 levels decreased upon MCC950 administration, sham as the NaHS-treated group (**Figures 6A–G**). These results indicate that NaHS can inhibit inflammasome activation in skin fibroblasts of diabetic rats.

### 3.7 Effect of NaHS on fibroblast pyroptosis in the skin wound tissues of diabetic rats

**Figure 7:**
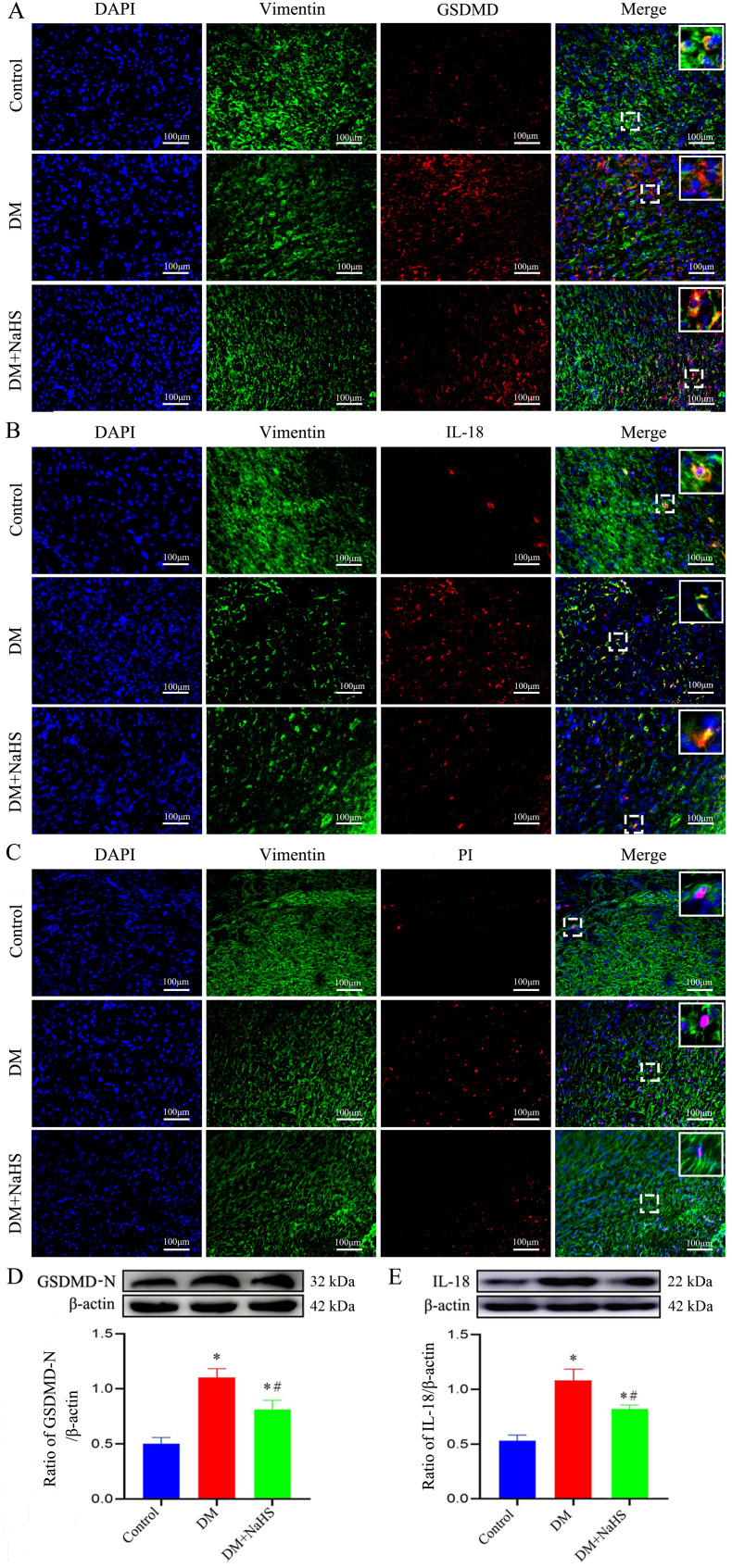
NaHS inhibits fibroblasts pyroptosis in skin wound tissues of diabetic rats. **(A-C)** Representative images of the colocalization of vimentin with GSDMD, IL-18, and propidium iodide (PI) in skin fibroblasts 21 days postoperatively. The yellow puncta in the merged images show the colocalization of vimentin with GSDMD, IL-18 and PI. Scale bar: 100 µm. **(D, E)** Representative western blots and quantitative analysis of the protein levels of GSDMD-N and IL-18 in skin wound tissues of the control, DM, and DM + NaHS groups 21 days postoperatively. *P < 0.05 vs. control group; ^#^ *P* < 0.05 vs. DM group; n = 6.

To determine the effect of NaHS on fibroblast pyroptosis in skin wound tissue, double immunofluorescent staining was used to detect the proteins expression of GSDMD and IL-18 (specific biomarkers for pyroptosis), which showed that GSDMD and IL-18 expressions were increased in the fibroblasts of the DM group as compared with those in the control group. However, this increase was reversed by NaHS treatment (**Figures 7A, B**). Similarly, western blotting analysis indicated that the administration of NaHS remarkably decreased the expression levels of GSDMD-N and IL-18 in the DM + NaHS group compared with those in the DM group (**Figures 7D, E**). In addition, propidium iodide staining revealed that the number of dead fibroblasts was higher in the DM group than in the control group. However, the administration of NaHS significantly attenuated the pyroptotic death of fibroblasts in skin wound tissues of diabetic rats (**Figure 7C**). These results indicate that NaHS can inhibit fibroblast pyroptosis in skin wound tissues of diabetic rats.

### 3.8 Effect of NaHS on NF-κB pathway in diabetic rats

**Figure 8:**
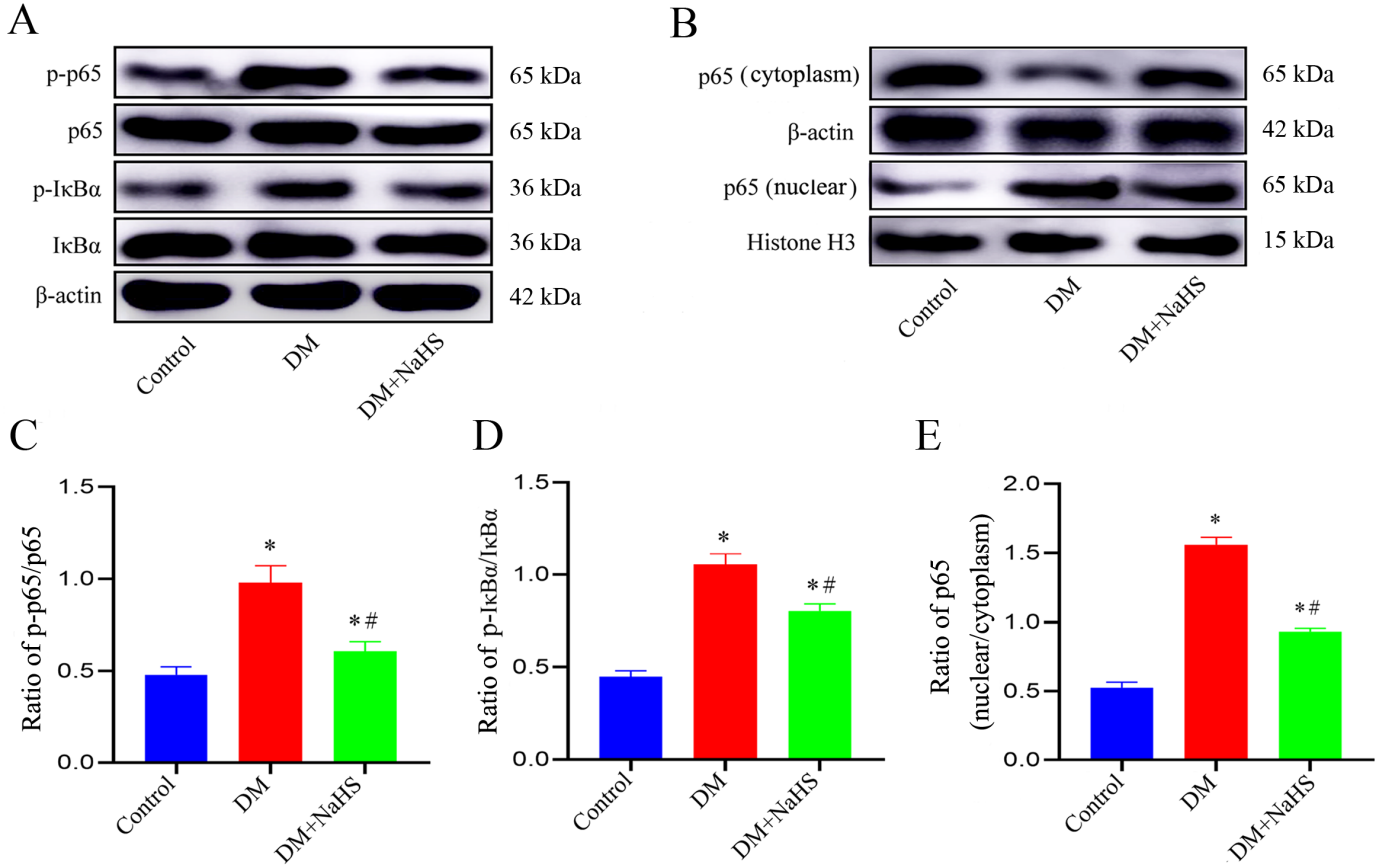
NaHS inhibits the NF-κB pathway activation in skin wound tissues of diabetic rats. **(A-E)** Representative western blots and quantitative analysis of the protein levels of p65, p-p65, IkBα, p-IkBα, and nuclear translocation of p65 in skin wound tissues of the control, DM, and DM + NaHS groups 21 days postoperatively. *P < 0.05 vs. control group; ^#^ *P* < 0.05 vs. DM group; n = 6.

To ascertain whether NaHS attenuated inflammation and pyroptosis in fibroblasts involved in regulation of NF-κB pathway, the phosphorylation levels of IκBα, NF-κB (p65), and the nucleus translocation of p65 were analyzed using western blotting. As shown in **Figures 8A–E**, the phosphorylation levels of p65 and p-IκBα were accentuated, and the p65 increased in cell nucleus of wound tissues from the DM group. However, administration of NaHS distinctly suppressed the phosphorylation of p65 and p-IκBα, and the translocation of p65 from the cytosol to nucleus. These results suggest that administration of NaHS can retard phosphorylation and nuclear translocation of the NF-κB pathway in skin wound tissues of diabetic rats.

## 4 Discussion

Chronic refractory wounds are one of the most serious complications of diabetes that severely diminish a patient’s quality of life and impose a heavy economic burden on patients, their families, society, and the health care system. Although numerous therapeutic approaches have been developed to facilitate the healing of diabetic wounds, such as dressing changes, surgical debridement, and cell therapies, effective therapies remain unavailable. In the present study, we demonstrated that NaHS treatment efficiently restored H_2_S levels in skin wound tissues and accelerated skin wound healing in STZ-induced diabetic rats, as evidenced by the promotion of collagen deposition and amelioration of histological changes. Moreover, our results demonstrated that NaHS treatment could inhibit macrophage M1 polarization, suppressed NLRP3 inflammasome activation, reduced inflammatory cytokine secretion, and attenuated fibroblast pyroptosis in skin wound tissues. Notably, we found that administration of NaHS was able to inhibit the activation of the NF-κB pathway in wound tissues of diabetic rats. Our findings suggest that H_2_S is a promising therapeutic agent for treating diabetic wounds.

H_2_S is a novel gaseous signaling molecule that is synthesized in mammalian tissues and plays a vital role in physiological processes and pathological conditions, including diabetes, stroke, and ischemic injury, in multiple organs and systems (25). Evidence suggests that plasma H_2_S level is significantly lower in diabetic patients (26). In addition, a previous study revealed that the H_2_S-synthesizing enzyme CSE and H_2_S level were substantially decreased in wound granulation tissue of ob/ob mice (27). In this study, we used an STZ-induced diabetic rat model to investigate the change in H_2_S content at wound sites and found that its level was remarkably reduced by diabetes, whereas administration of NaHS effectively elevated H_2_S levels in the wound tissues. However, the mechanism whereby H_2_S level is downregulated in diabetic skin wound tissues remains unclear. We speculate that hyperglycemia negatively influences the activity and/or expression of H_2_S synthesizing enzymes, and that NaHS application may ameliorate these alterations by up-regulating CSE, CBS, and 3MST protein expressions in diabetic wound tissues, suggesting a sustained NaHS effect on the H_2_S biosynthesis pathways throughout the wound healing process. Our previous study revealed that NaHS could promote placental angiogenesis in cigarette smoke exposure rats (28). The results of this study indicated that NaHS increased blood supply to the wound, suggesting that exogenous H_2_S administration might accelerate angiogenesis in the diabetic skin wound, consisting with the findings that H_2_S could stimulate angiogenesis under both physiological and diabetic conditions (29, 30).

As expected, NaHS treatment markedly promoted skin wound healing in diabetic rats. The effect of NaHS was glucose independent, as it did not affect glucose levels, and there was no apparent difference in rat body weight between the DM and DM + NaHS groups. Wound healing is a complex biological process involving multiple cell types, of which fibroblasts are key cells that maintain tissue rebuilding and facilitate wound closure. Fibroblasts are also important for extracellular matrix synthesis, collagen deposition, wound contraction, and tissue remodeling (31). Although no data indicated directly that NaHS affected fibroblast functional properties, we found that NaHS treatment inhibited fibroblast pyroptosis and promoted collagen deposition, including collagen types I, II, III, and IV in the wound tissues of diabetic rats compared to those in the DM group, which is in line with the results of a previous investigation showing that collagen deposition facilitated proper tissue integration and helped skin wound healing in diabetic rats (4). There is evidence that H_2_S is involved in the mediation of cell proliferation, differentiation, migration, cell-matrix adhesion, and extracellular matrix assembly (32); this may partially explain the effect of NaHS in regulating fibroblasts to produce more collagen and facilitate wound healing in diabetic rats.

Numerous studies have shown that inflammation plays a crucial role in wound healing. A proper inflammatory response is essential for wound healing; however, exacerbated and persistent inflammation hinders wound healing and results in scar formation during wound closure (33). Accumulating evidence has shown that aberrant polarization of M1 and M2 macrophages at wound sites leads to a delay in wound closure (34). Thus, interventions promoting a shift of macrophages from the proinflammatory M1 to the anti-inflammatory M2 phenotype may have therapeutic potential for the treatment of diabetic skin wounds. To investigate the effect of NaHS on macrophage polarization, we detected the markers of M1 and M2 macrophages by double-immunofluorescence staining in the skin wound tissues of diabetic rats. These results indicated that NaHS treatment promoted the transformation of the M1 to M2 phenotype. The results of western blotting, for the protein levels of M1 phenotype (iNOS and CD80) and M2 phenotype (CD206) protein expression were consistent with the results of immunofluorescence staining. Notably, our findings suggest that NaHS promotes the skewing of macrophages towards the M2 subtype, which may be involved in the promotion of healing in diabetic skin wounds. Hyperglycemic environment in diabetic wound reportedly triggers macrophages to secrete proinflammatory cytokines (e.g., TNF-α, IL-1β, and IL-6), thereby inducing M1 macrophage polarization and chronic inflammation (35, 36). Moreover, hypoxia endothelial cells-derived exosomes reportedly accelerated diabetic wound healing by improving endothelial cell function and promoting M2 macrophage polarization (37). Consistent with this notion, our results showed the levels of pro-inflammatory cytokines including TNF-α, IL-β, and IL-6 in skin wound tissues of the diabetic rats were apparently increased compared to that of the non-diabetic rats; however, administration of NaHS remarkably reduced these cytokines, suggesting that NaHS exerts anti-inflammatory effects partly due to its suppression of macrophage M1 polarization and the reduced release of pro-inflammatory cytokines.

Evidence from multiple sources indicates that inflammasomes are involved in the inflammatory process and are critical contributors to delayed wound healing in diabetes (38). As an essential component of the immune system, the NLRP3 inflammasome acts as an intracellular sensor of a variety of endogenous, exogenous, and pathogenic signals. Under the stimulation of danger signals, NLRP3 proteins interact with ASC and activate caspase-1, result in the maturation and release of cytokines, such as IL-1β and IL-18, and induce cells pyroptosis (39). Previous studies have indicated that H_2_S decreases NLRP3 protein expression, attenuates cardiac dysfunction and injury in diabetic rats (40), and ameliorates diabetes-associated cognitive dysfunction via regulation of the nuclear factor erythroid 2-related factor 2/heme oxygenase-1 (Nrf2/HO-1) axis and the NLRP3 inflammasome pathway in diabetic rats (41). In accordance with these findings, the results of this study showed a marked increase in the protein expression of NLRP3 inflammasome components ASC, NLRP3, and cleaved caspase-1 in diabetic wound tissues. However, after treating diabetic rats with NaHS, the levels of NLRP3 inflammasome-associated proteins were reduced in the wound tissues. To further validate how NaHS affects NLRP3 inflammasome activation, we used MCC950 as a comparative agent and observed similar results between the MCC950 and NaHS treatment groups, suggesting that H_2_S might exert its anti-inflammatory effect through NLRP3 inflammasome modulation. Several recent studies have revealed that NLRP3 inflammasome-mediated pyroptosis is observed in the cerebral ischemia–reperfusion model (42) and in ionizing radiation-induced lung injury (43). A recent study showed that H_2_S ameliorates lipopolysaccharide-induced depression-like behavior by suppressing the NLRP3 inflammasome and pyroptosis in the hippocampus of mice (44). In the present study, immunofluorescence staining and western blotting analysis demonstrated that NaHS treatment robustly reduced the levels of pyroptosis-associated proteins, including GSDMD and cleaved caspase-1, in the fibroblasts of diabetic wound tissues, which is in accordance with a previous study (45), suggesting that the protective effect of NaHS may be related to the inhibition of pyroptosis in fibroblasts.

**Figure 9:**
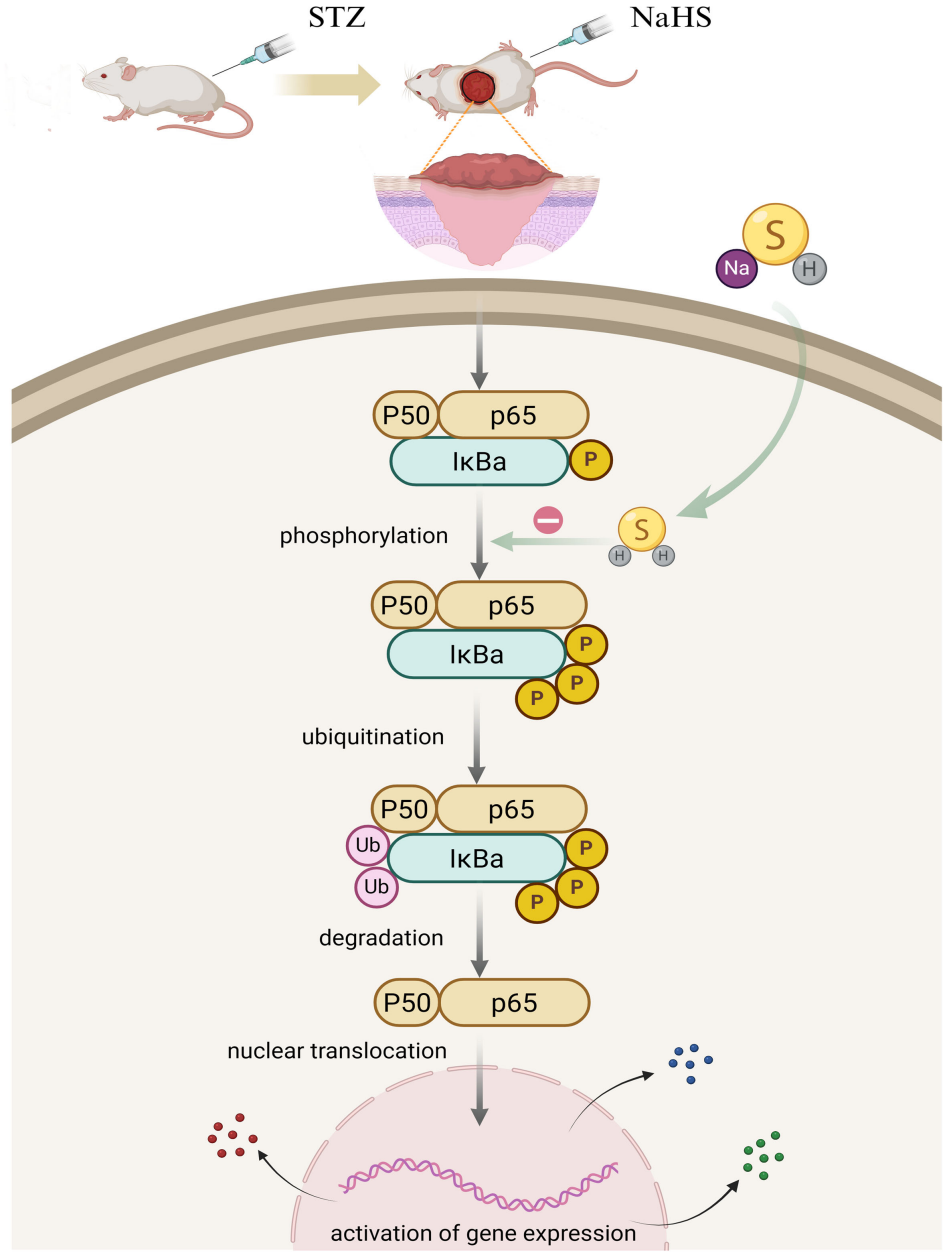
Putative model to elucidate the role of H_2_S in NF-ĸB signaling pathway regulation in the skin wound of diabetic rats. In diabetic wounds, various factors trigger NF-κB pathway activation, resulting in a cascade of events including IκBα phosphorylation, ubiquitination, and degradation, followed by the translocation of free NF-κB dimers into the nucleus. Hypothetically, H_2_S exerts anti-inflammatory effects by inhibiting NF-κB signaling pathway activation in diabetic wounds.

Phosphorylation of the NF-κB pathway induced by various pathological stimuli, such as lipopolysaccharide (46), ischemia–reperfusion (47), and alcohol (48) is a prerequisite for the transcriptional activation of NLPR3 inflammasome. Studies have shown that H_2_S regulates the toll-like receptor 4 (TLR4)/NF−κB pathway and attenuates cigarette smoke induced pyroptosis (49), and ameliorates chronic renal failure via suppression inflammation and apoptosis by modulating ROS/mitogen-activated protein kinase (MAPK) and NF-κB pathways in rats (50). Based on these findings, we explored whether NaHS could regulate the NF-κB pathway in diabetic wound tissue. NaHS treatment markedly suppressed NF-κB pathway activation as indicated by decreased phosphorylated of NF-κB p65 and IκBα in skin wound tissues of the diabetic rats. Furthermore, we found that NaHS could inhibit the translocation of free NF-κB p65 into the nucleus and reduce pro-inflammatory cytokines in diabetic wound tissue. These results suggest that inhibition of the NF-κB pathway may be a critical mechanism responsible for the effect of NaHS in alleviating inflammation and facilitating skin wound healing in diabetic rats (**Figure 9**). Consistent with our findings, several recent studies report that H_2_S reduces lipopolysaccharide- (51), high glucose- (52), high fat- (53), and uranium-induced (54) inflammation via inhibition of the NF-κB pathway.

This study retains certain limitations. Importantly, we used only a single NaHS dose to determine how H_2_S affects NLRP3 inflammasome activation and fibroblast pyroptosis in the skin wound tissues of diabetic rats. Therefore, dose-dependent effects should be considered in future studies. According to our data, NaHS administration can significantly promote diabetic wound healing. However, limitations restrict NaHS application in clinical settings due to the fast and uncontrollable H_2_S release. To better mimic endogenous H_2_S production and release conditions, a slow-releasing H_2_S donor, or H_2_S delivery platform, such as a multifunctional hydrogel, should be considered in future studies. The present study demonstrated that the NF-κB pathway might contribute to alleviating inflammation and promoting skin wound healing in diabetic rats upon NaHS treatment. However, whether other molecular pathways are involved in the regulatory mechanisms of H_2_S should be considered.

In summary, our findings uncovered a novel role for H_2_S in diabetic skin wound healing, which regulates NLRP3 inflammasome activation and fibroblast pyroptosis, suggesting that H_2_S may be a promising therapeutic agent for treating diabetic skin ulcers.

## Data Availability

The original contributions presented in the study are included in the article/supplementary material. Further inquiries can be directed to the corresponding author.
